# Segmentectomy as a safe and equally effective surgical option under complete video-assisted thoracic surgery for patients of stage I non-small cell lung cancer

**DOI:** 10.1186/1749-8090-8-116

**Published:** 2013-04-29

**Authors:** Xiaojing Zhao, Liqiang Qian, Qingquan Luo, Jia Huang

**Affiliations:** 1 Shanghai Lung Cancer Center/Shanghai Chest Hospital, 241 West HuaiHai Road, Shanghai, China

## Abstract

**Background:**

While video-assisted thoracic surgery lobectomy has been widely accepted for the treatment of non–small cell lung cancer, the debate over video-assisted thoracic surgery segmentectomy still remains. This study compared the clinical outcomes using the two procedures for stage I non–small cell lung cancer patients.

**Methods:**

Retrospective review was conducted on patients who underwent video-assisted thoracic surgery segmentectomy or lobectomy for clinical stage I non–small cell lung cancer at Shanghai Chest Hospital between November 2009 and May 2012. Video-assisted thoracic surgery segmentectomy was performed on 36 patients and video-assisted thoracic surgery lobectomy on 138 patients. Comparisons between the 2 groups were performed in patient demographic and clinical characteristics, intraoperative parameters and oncology outcomes.

**Results:**

Mean volume of chest tube drainage after operation was smaller for segmentectomy than for lobectomy (1021 ml vs. 1328 ml, P=0.036). Other parameters analysis including blood loss, operation time, chest tube duration and length of hospital stay favors the segmentectomy group numerically without significance. There was no significant difference in distributions in both intra and post operative complications. There was one peri-operative mortality from segmentectomy group and all other patients are alive with a median follow up of 327 days. There were 1 (2.8%) locoregional recurrence after segmentectomy and 6 recurrences (4.4%) after lobectomy (P=1.00). Multivariate survival analysis revealed no significant difference in recurrence-free survivals between the two groups. Two patients successfully underwent bilateral segmentectomies and are free of disease.

**Conclusions:**

For patients with stage I non–small cell lung cancer, video-assisted thoracic surgery segmentectomy offers a safe and equally effective option and can be applied to complicated operation such as bilateral segmentectomy.

## Background

Non-small cell lung cancer (NSCLC) represents approximately 80% of all lung cancers [1] and the traditional treatment for stage I NSCLC is lobectomy. In 1973, Jensik et al [2] published a study suggesting that segmental resection was equivalent to lobectomy and represented an adequate operation for stage I NSCLC. This publication started a debate regarding the optimal surgical approach for early stage NSCLC. Recently, as a result of an increasing incidence of small lung tumors, there has been renewed interest in the use of anatomic segmentectomy, especially for patients with stage IA NSCLC and those unable to tolerate lobectomy because of compromised medical condition. Several recently published studies have shown that segmentectomy could be performed safely without compromising oncologic results if patient selection for sublobar resection was adequate [3-8].

Video-assisted thoracic surgery (VATS) has been introduced in a variety of thoracic operations, including small-sized lung cancer. VATS is less invasive and provides benefits due to decreased postoperative pain, shorter recovery, decreased inflammatory response and improved tolerance of chemotherapy [9-16]. VATS can be generally classified into two categories of Hybrid-VATS and Complete-VATS (C-VATS). C-VATS, the pure video-based operation was the procedure we used in our studies.

Despite the growing acceptance of VATS lobectomy, VATS segmentectomy, especially C-VATS segmentectomy has not become widespread and remains highly controversial as a choice for treatment of small lung tumors. In addition to concerns about increased locoregional recurrence, potential arguments against VATS segmentectomy include higher rates of complications and inadequate nodal dissection because of the high complexity of the procedure [5,17]. To date, there is scanty of data from published studies comparing clinical outcomes between VATS segmentectomy and VATS lobectomy except the data from Japan and US groups on selected patients [1,18]. The purpose of this study was to offer a comprehensive evaluation of the clinical outcomes of complete VATS segmentectomy compared with VATS lobectomy in stage I NSCLC patients under varying degree of surgical complexity.

## Methods

### Patients

In this retrospective study, 174 stage I NSCLC patients were operated on at the Shanghai Chest Hospital (Shanghai, China) between November 2009 and May 2012. VATS segmentectomy was performed on 36 patients, and VATS lobectomy on 138 patients. Both procedures were accompanied by systemic lymphadenectomy. Principally, all patients were operated with curative intent under C-VATS.

The hospital center board of management approved the conduct of this study including a waiver of individual patient consent during data collection due to the retrospective nature of the study. Inpatient and outpatient charts were reviewed, and all the data including demographic data, histopathologic categorization, pathologic staging, number of nodal stations resected, number of lymph nodes resected, operative courses, intraoperative blood loss, total volume of chest tube drainage after operation, chest tube duration, postoperative hospital courses and information of recurrence were collected with patients’ consent. Patients were contacted for additional follow-up information with a standardized questionnaire every 6 months (or every 3 months when necessary). Operative mortality was defined as death within 30 days of the procedure. Pathologic cancer staging was done in accordance with TNM-7 for the staging of NSCLC (2009).

Before surgical resection, all patients underwent preoperative examinations including ECG, chest X-rays, abdominal ultrasound, pulmonary function test, chest CT scanning (3D reconstruction when necessary), enhanced head MRI and bone scanning. Patients selected for segmentectomy must fulfill the following criteria: 1) compromised pulmonary reserve with extensive comorbidities, and without the ability to endure lobectomy, 2) peripheral location of the tumor with the biggest diameter ≤ 2 cm, and bronchioloalveolar carcinoma (BAC) simplex and/or ground-glass opacity of nodules ≥ 50% as shown by CT scanning, and 3) Tumor doubling time (TDT) ≥ 400 days as demonstrated by follow-up imaging examination. Patients selected for lobectomy must fulfill the following criteria: 1) patients with no anatomic or surgical contraindications; 2) No compromise of standard oncologic and dissection principles of thoracic surgery.

**Figure 1:**
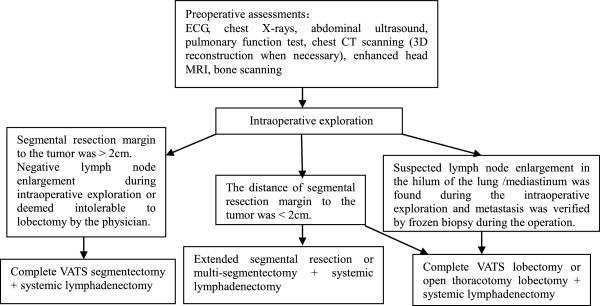
Standard preoperative evaluation and algorithm of assessments and procedures.

The standard preoperative evaluation and algorithm of segmentectomy assessments and procedures are shown in Figure 1. Patients whose operation converted to thoracotomy were considered censored on the date of operation when survival analysis was performed and were part of the intent-to-treat population.

### Selection of incisions: 3-ports or 4-ports approach

Operation technique

**Observation port **It is typically at midaxillary line and the 7-8^th^ intercostal space about 0.5-1 cm in length. It is primarily used for placing trocar lens of thoracoscope into thoracic cavity, and reserved for the placement of upper chest tube.

**Main operation port **It is generally placed at the intersection of anterior axillary line and the 4^th^ intercostal space and about 2–5 cm in length. The use of an incision protection sleeve will protect the incision from tumor-seeding. The size and position of the port can be adjusted according to the tumor. After the operation, the port will be sutured.

**First accessory operation port **It is usually placed at the intersection of posterior axillary line and the 8-9^th^ intercostal space. It can be positioned visually with the aid of a thoracoscope, thus avoiding the operation inconvenience and postoperative pain by not causing injury or touching the diaphragm. This port is usually used for traction and exposition of the tissues and for the placement of anastomat and ultrasound knife into thoracic cavity. Since it is near the costophrenic angle, it is usually reserved for the placement of the lower chest tube after the operation.

**Second accessory operation port **It is usually placed at the intersection of posterior axillary line and the 5-6^th^ intercostal space, or closer to the subscapular angle line. It may not be needed if the operation is simple. This port is mainly used for placing some instruments such as ovalforceps and pulmonary forceps into the thoracic cavity to expose tissues. It will be sutured after operation.

### Operative procedures for VATS segmentectomy

**Exploration and identification **Generally, specific position of tumor can be accurately located by CT scanning or three dimensional reconstructions before operation. If necessary, the tumor can also be located by hook-wire puncture preoperatively [19]. During the operation, the position of the tumor is reconfirmed via finger palpation.

**Resection margins to the tumor **After the location of the tumor had been confirmed, we also used an electric knife (argon gas) to provide a contour of the margins. This was further assisted by segmental lung inflation. In case the estimated distance to the margins is shown to be less than 2 cm, use of original margins (> 2 cm) would still be upheld in order to completely resect the tumor.

**Handling of important lung segments **All patients of the VATS segmentectomy group received the anatomic resection with the arteries, veins and bronchial structure of targeted lung segments completely isolated and processed as described in previous reports [15].

**Handling of segmental fissure /lung tissue **Generally, there are no obvious fissures between two segments. Moreover, the tissue of lung is relatively thick and considerations should be taken when choosing the appropriate anastomat.

The specimens are usually taken out of thoracic cavity from the main operation port after being placed into an aseptic bag. This is followed by immediate exploration. It should be noted that the distance of segmental resection margin to the tumor must be ≥ 2 cm. If the distance is < 2 cm, extended segmentectomy or pulmonary lobectomy, will have to be considered. In our series of patients, we have been consistent and stick to the rule that the surgical margins must be ≥ 2 cm as the margin has been considered necessary to avoid relapse. We did not have patients whose surgery had to be compromised for this rule.

After being rinsed and soaked with aseptic distilled water, the thoracic cavity is carefully examined by the surgeon to identify any active bleeding. Afterwards, the samples are sent for frozen section pathology examination.

### Operative procedures for VATS lobectomy

Up to now, convention has already been established basically for VATS lobectomy depending on specific situation such as interlobar fissure. The commonly used methods include RJ.Mckenna’ sequential thoracoscopic lobectomy [20], Liu’s single-direction thoracoscopic lobectomy [21] and Wang’s thoracoscopic lobectomy [22].

### Lymph nodes dissection

If lung cancer is confirmed by intraoperative frozen section, systematic lymphadenectomy will be performed for both VATS segmentectomy and VATS lobectomy according to the commonly accepted international guidelines. After the lymphadenectomy, “skeletonized” is achieved for important structures. Additionally, parabronchial lymph nodes, segmental lymph nodes and sub-segmental lymph nodes (groups 12, 13 and 14) are required to be dissected as thoroughly as possible for VATS segmentectomy.

### Hemostasis

Hemostasis is performed carefully after the lymph nodes dissection, and the thoracic cavity is rinsed and soaked with aseptic distilled water again. Then the lung is inflated by an anesthetist again to make sure that there is no air leakage at the residue of bronchus. To reduce drainage volume after lymphadenectomy, the spaces left are padded with hemostatic gauze. Two chest tubes (the upper and the lower) are usually kept after operation.

## Statistical analysis

All statistical analysis was performed using SAS version 9.2 (SAS institute Inc., USA). The t-test was used for continuous data, and Chi-square test was used for categorical data. Recurrence-free survival was estimated with the Kaplan-Meier method and evaluated by log-rank test. Recurrence-free survival was defined as the time from surgery to the first diagnosis of local, regional, or distant disease recurrence or until the last follow-up. Cox regression analysis was used to quantify hazard ratios of treatment procedures with respect to recurrence-free survival while allowing for significant covariates. Statistically significant differences were set as P < 0.05. Based on the results of univariate analysis, significant covariates of tumor size, tumor stage and histology were next chosen in the Cox model. Here an all-factor model was used to show hazard ratios of the two procedures allowing for these covariates.

## Results

### Demographic and clinical characteristics of the patients

During the stated period, 174 patients with stage I NSCLC underwent complete VATS in our hospital. VATS segmentectomy was performed on 36 patients, and VATS lobectomy on 138 patients. Both procedures were accompanied by systemic lymphadenectomy. One patient in segmentectomy group died of cerebral infarction 7 days post operation, and his death had no relationship with the procedure as judged by the investigators. The 30-day mortalities were 2.8% and 0.0% in VATS segmentectomy group and VATS lobectomy group, respectively. Clinicopathologic factors are shown in Table 1. Stage IA comprised 137 patients, and stage IB 37. Their ages ranged from 36–81 years with a mean of 59.3 years for segmentectomy, and 35–81 years with a mean of 58.8 years for lobectomy. The two groups had similar distribution in age and sex. However, mean tumor size (maximum tumor diameter) in the VATS segmentectomy group was significantly smaller than in the VATS lobectomy group (14.2 vs. 19.8 mm, P = 0.002; Table 1). The distribution of stage IA and IB was also significantly different between segmentectomy and lobectomy (P = 0.001). The histopathology distribution was different between segmentectomy and lobectomy (P = 0.031), with adenocarcinoma (ADC) and bronchiolo-alveolar carcinoma (BAC) accounting for 61.1% and 25.0% in the segmentectomy group, while in lobectomy group, the percent of ADC was predominantly higher (80.4%). The localization of the tumor was 110 (18 for segmentectomy and 92 for lobectomy) in the right lung and 66 (20 for segmentectomy and 46 for lobectomy) in the left lung. 3 patients in our study successfully underwent multiple segmentectomies and 2 of them underwent bilateral segmentectomies as their bilateral primary lesions were found in both lungs. They are free of recurrence and well now. A list of resected segments is shown in Table 2.

**Table 1:** Demographic and clinical characteristics

**Charecteristics**	**Segmentectomy (N=36)**	**Lobectomy (N=138)**	**Total (N=174)**	**P**
**Age (years, mean ± SD)**	59.3 ± 13.77	58.8 ± 10.77	58.9 ± 11.41	0.82
**Sex (male/female)**	12:24	52:86	64:110	0.70
**Tumor size(mm, mean ± SD)**	14.2 ± 7.15	19.8 ± 9.82	18.6 ± 9.58	0.002
Histology (No, %)*				0.031
ADC	22 (61.1%)	111 (80.4%)	133 (76.4%)	
BAC	9 (25.0%)	20 (14.5%)	29 (16.7%)	
others	5 (13.9%)	7 (5.1%)	12 (6.9%)	
**Stage (No, %)**				0.001
IA	35 (97.2%)	102 (73.9%)	137 (78.7%)	
IB	1 (2.8%)	36 (26.1%)	37 (21.3%)	

P<0.05 is considered statistically significant.

* *ADC*:adenocarcinoma, *BAC*:bronchioloalveolar carcinoma.

**Table 2:** Tumor location

	**Lobectomy(N=138)**	**Segmentectomy(N=36)**
**Left lower lobe**	15	
S6		8
**Left upper lobe**	31	
S1+2		1
S1+2+3		8
S4+5		3
**Total left lobe**	46	20
**Right lower lobe**	26	
S6		7
**Right lower middle lobe**	1	
**Right middle lobe**	12	
**Right upper lobe**	52	
S1		7
S2		4
**Right upper middle lobe**	1	
**Total right lobe**	92	18

Note: lesions from different segments of same patient are counted separately.

### Intraoperative parameters and complications

Total mean volume of chest tube drainage after operation were smaller for segmentectomy than for lobectomy (1021 ml vs. 1328 ml, P=0.036). Other parameters including blood loss, operation time, chest tube duration and length of hospital stay favor the segmentectomy group numerically without significance (Table 3).

**Table 3:** Intraoperative parameters

**Parameters**		**Segmentectomy (N=36)**	**Lobectomy (N=138)**	**Total (N=174)**	**P**
**Operation time (h)**	Mean (S.D)	124.8 (45.29)	127.2 (35.16)	126.7 (37.35)	0.73
	Median	112.0	122.5	119.5	
	Min, Max	75, 271	67, 232	67, 271	
**Intraoperative blood loss (ml)**	Mean (S.D)	162.5 (257.84)	180.4 (201.86)	176.7 (213.94)	0.66
	Median	100.0	100.0	100.0	
	Min, Max	50, 1600	50, 2000	50, 2000	
**Total volume of chest tube drainage after operation (ml)**	Mean (S.D)	1021.4 (591.72)	1328.9 (819.34)	1264.9 (785.96)	0.036
	Median	810.0	1030.0	1000.0	
	Min, Max	310, 2540	350, 4890	310, 4890	
**Chest tube duration (day)**	Mean (S.D)	4.1 (1.41)	4.5 (1.78)	4.4 (1.72)	0.14
	Median	4.0	4.0	4.0	
	Min, Max	2, 8	2, 12	2, 12	
**Postoperative hospital stay (day)**	Mean (S.D)	6.2 (1.62)	6.5 (1.87)	6.4 (1.82)	0.38
	Median	6.0	6.0	6.0	
	Min, Max	4, 11	4, 14	4, 14	

Intraoperative bleeding was the only reason of conversions to minithoracotomy for one patient in the segmentectomy group (2.8%) and six patients in the lobectomy group (4.3%) as seen in Table 4, respectively. For the patient under segmentectomy, his hemostasis was achieved by video-assisted angiorrhaphy during the operation. 1500 ml of blood was given to this patient during the surgery; however, he did not have any intraoperative hemodynamic instability. These conversions were assessed as unrelated to the procedure under study as judged by the investigators.

**Table 4:** Intraoperative and postoperative complications

		**Segmentectomy (N=36)**	**Lobectomy (N=138)**	**Total (N=174)**	**P**
		**n (%)**	**n (%)**	**n (%)**	
**Intraoperative complication**	hemorrhoea	1 (2.8%)	6 (4.3%)	7 (4.0%)	
	pleural adhesions	-	4 (2.9%)	4 (2.3%)	
	total	1 (2.8%)	10 (7.2%)	11 (6.3%)	0.85
**Postoperative complication**	pneumoderma	1 (2.8%)	1 (0.7%)	2 (1.1%)	
	air leakage	-	1 (0.7%)	1 (0.6%)	
	hemorrhoea	-	1 (0.7%)	1 (0.6%)	
	hypoxemia	2 (5.6%)	-	2 (1.1%)	
	total	3 (8.3%)	3 (2.2%)	6 (3.4%)	0.07

*P<0.05 is considered statistically significant.

Specific intraoperative and postoperative complications after each procedure are listed in Table 4. During the operation, there was 1 case of hemorrhoea in VATS segmentectomy group, 6 cases of hemorrhoea and 4 cases of pleural adhesions in VATS lobectomy group. The incidence rates of intraoperative complications were not significantly different (2.8% versus 7.2%, P = 0.80). As for postoperative complications, the incidence difference between groups was not significant, either (8.3% versus 2.2%, P = 0.07). There were 1 case of pneumoderma and 2 cases of hypoxemia in VATS segmentectomy group, and 1 case of pneumoderma, 1 case of air leakage and 1 case of hemorrhoea in VATS lobectomy group.

### Local recurrence, distant metastases, and recurrence-free survival

**Figure 2:**
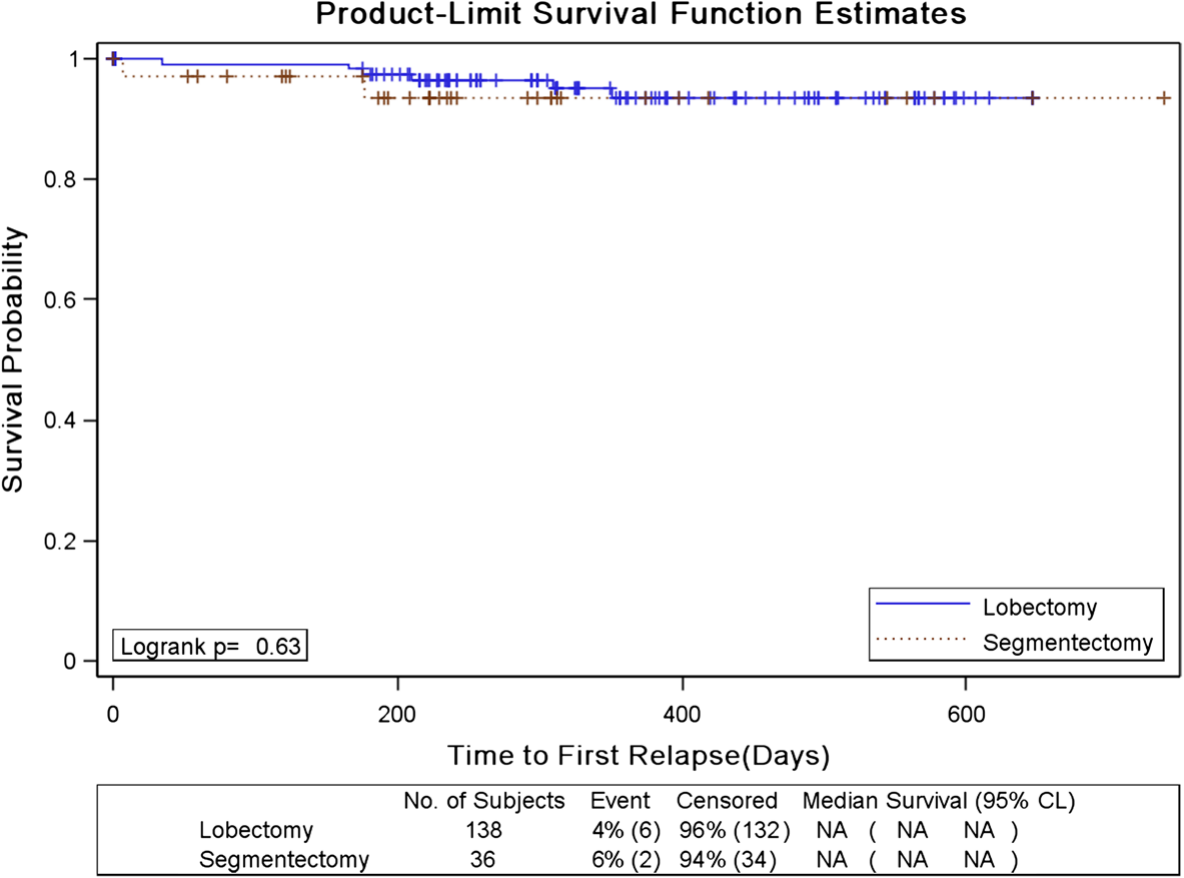
Recurrence-free survival.

As shown in Table 5, 1 (2.8%) of 36 segmentectomy patients and 6 (4.4%) of 138 lobectomy patients relapsed during the follow-up period with a median follow up of 327 days and the difference was not significant (P= 1.00). The locoregional recurrence rate was low in both groups, with 1 case (2.8%) in VATS segmentectomy group and 3 (2.2%) in the lobectomy group. Up to date, there are no cancer related mortalities in both groups. Furthermore, recurrence-free survivals were also similar between the groups (**P=0.63**) and numerically favored the segmentectomy group (Table 6, Figure 2). Multivariate analysis that had taken tumor staging, tumor size and histopathological classification into consideration also showed no significant difference in the rate of recurrence between two types of resection (Table 6).

**Table 5:** Local recurrence and distant metastases

**Characteristics**		**Segmentectomy (N=36)**	**Lobectomy (N=138)**	**Total (N=174)**	**P**
		**n (%)**	**n (%)**	**n (%)**	
**Recurrences**	distant	0	3 (2.2%)	3 (1.7%)	1.00
	locoregional	1 (2.8%)	3 (2.2%)	4 (2.3%)	1.00
	total	1 (2.8%)	6 (4.4%)	7 (4.0%)	
**Distant metastases**	brain and bone metastases	0	1 (0.7%)	1 (0.6%)	1.00
	bone metastasis	0	1 (0.7%)	1 (0.6%)	
	pericardial effusion	0	1 (0.7%)	1 (0.6%)	
	total	0	3 (2.1%)	3 (1.8%)	
**Local recurrences**	offside	1 (2.8%)	1 (0.7%)	2 (1.1%)	0.61
	ipsilateral	-	2 (1.4%)	2 (1.1%)	
	total	1 (2.8%)	3 (2.2%)	4 (2.2%)	

*P<0.05 is considered statistically significant.

**Table 6:** Multivariate analysis of factors predicting recurrence among patients with stage I non–small cell lung cancer

***Risk factor***	***HR***	***95% CI***	***P***
**Segmentectomy versus lobectomy**	0.52	0.10~2.74	0.44
**tumor sizes**	1.19	0.47~3.00	0.72
**tumor stage**	0.86	0.12~6.03	0.88
**histology**			
BAC versus AD	0.00	0.00~	1.00
OTHER versus AD	0.00	0.00~	1.00

*ADC*: adenocarcinoma, *BAC*: bronchioloalveolar carcinoma.

## Discussion

The debate over whether segmental resection was equivalent to lobectomy as an adequate operation for stage I NSCLC can be dated back to 1973 [2]. The results of the first randomized trial were published by the Lung Cancer Study Group (LCSG) in 1995 where sublobar resection was shown to have a three-fold increase of local recurrence relative to lobectomy [23]. However, reports almost two decades later have showed that anatomical segmentectomy provided compatible results in the recurrence rates and survival if adequate selection (stage IA and IB) was performed for NSCLC [3,24]. Okada et al. compared the results of anatomical segmentectomy with lobectomy and wedge resection in small-sized NSCLC [25]. The 5-year cancer-specific survivals of patients with pathologic stage I disease with tumors of 20 mm or less in diameter were 92.4% after lobectomy, 96.7% after segmentectomy, and 85.7% after wedge resection, respectively. Another study indicated a lack of difference between segmentectomy and lobectomy in patients with tumors less than 2 cm in diameter [26]. These results support segmentectomy as one of the operative options to cure small NSCLC. Our study is the only one to date to provide a comprehensive comparison on peri-operative and post- operative morbidity, recurrence and survival between VATS segmentectomy and VATS lobectomy among Chinese patients.

Although the use of the VATS procedure for lung cancer has increased in the past several years, and minimally invasiveness has been shown in previous reports [27], most of these procedures were applied for lobectomy and wedge resection, but rarely segmentectomy. The hesitancy of using VATS segmentectomy may be attributed to surgical complexity, concerns regarding increased morbidity related to prolonged air leak and local recurrence rates. To date, there are few publications on VATS segmentectomy in small lung cancers except for Japanese and American groups among highly selected patients [1,18] and none has been reported among Chinese patients.

Selection of patients following the criteria of NCCN [28] proved to be an important factor in our successful operations. In addition, the location of the tumor needs to be determined precisely using CT or other assisted technologies as according to the plan. This will guide the choice whether to pursue segmentectomy next. Based on our experience, single segmentectomy may not be sufficient for those tumors located in the intersections of several segments as the resection margin to the tumor cannot be guaranteed and thus the risk of recurrence may increase. Consequently, multi-segmentectomies or lobectomy is obviously called for in these cases to achieve the purpose to eradicate of the disease.

Due to its underlying structure of the artery and bronchus, not every segment is amenable to anatomic resection. Those segments which are generally held to be resectable are located in the apical right upper segment, posterior segment, dorsal right lower segment, posterior left upper segment plus anterior (inherent upper), the lingual and left posterior dorsal segments. Anterior upper segment was anatomically considered difficult to resect and we were able to successfully perform the operation on one patient. We agreed that composite basilar segment is the most difficult one to perform anatomic resection [29].

Extreme care should also be taken when addressing the parenchymal surgical margins for segmentectomy. After dividing the blood vessels and segmental airway that tether the lung, focus will be on determining the resection margin to the primary tumor. Among factors such as inflation of clapped bronchus and injection guided by bronchoscopy to determine the resection margin, the physical distance to the margin was considered to be most important factor to reduce relapse for segmentectomy patients [30]. 2 cm margin has been considered necessary to avoid relapse [29]. The low recurrence rates we observed in our study may also be attributed to the conservative approach adopted when determining resection margins to the tumor.

For total volume of chest tube drainage after operation, the VATS segmentectomy group presented with smaller amount of drainage compared with VATS lobectomy group. This may be attributed to the relatively small cavity left after segmentectomy, and can soften the postoperative distress and lead to earlier recovery of patients after the procedure. For the remaining intraoperative parameters including operation time, intraoperative blood loss, chest tube placement duration and postoperative hospital stay, there were no significant differences between the 2 groups. Furthermore, the rates of both intraoperative (2.8% versus 7.2%, P > 0.05) and postoperative complications (8.3% versus 2.2%, P > 0.05) were not significantly different between segmentectomy and lobectomy. The lack of significant difference was consistent with a previously published report [18]. However, our rates are much lower than those in other reports, in which the average rates of postoperative complications were 17.6% to 31.3% after VATS segmentectomy, 32% to 39% after open segmentectomy, and 15.3% to 23.8% after VATS resection [9,15,24,31,32]. The explanation for the gap of rates may well be that only severe cases were counted in our study and improvement of techniques and knowledge over time.

In the last decade, a growing number of published studies have demonstrated that segmental resections achieve comparable oncologic and survival outcomes to lobectomies [3-7,24]. Schuchert et al. [3] published results of 182 segmentectomies (done with thoracotomy and VATS) and 246 lobectomies in patients with NSCLC stage IA and IB. Similar overall recurrence rates were observed after segmentectomy (17.6%) and lobectomy (16.7%). Again, similar recurrence rates between segmentectomy and lobectomy were reported by Shapiro et al. [18] (overall recurrence rates: 17.2% versus 20.4%) while Yamashita et al. [1] reported lower rates (locoregional recurrence rates: 7.9% versus 5.6%; distant metastasis rates: 5.3% versus 5.6%). In our study, the recurrence rates were lower than the above published data. Our overall recurrence rate in the VATS segmentectomy group was 2.8%, versus 4.4% in the VATS lobectomy group. Specifically, the locoregional recurrence rates and distant metastasis rates were 2.8% and 0.0% for VATS segmentectomy and 2.2% and 2.2% for VATS lobectomy, respectively. Our low recurrence rates are halved when comparing to those of Japanese group as we have roughly followed up patients half as long (average of 327.0 days versus 27.5 months). Again, our lower recurrence rates as well as those reported in Japan strongly suggest the wider acceptance of VATS technique and operational improvement of our thoracic surgeons over the past decade.

Sienel et al. [33] pointed that the frequency of local recurrence following segmentectomy might be influenced by segment localization, and segmentectomy within the S1-3 region which may have some relations with the high local recurrence rate. The only observed local recurrence after segmentectomy in this study occurred in the S1-2 region and is consistent with Sienel’s results.

We found no significant differences of recurrence between the 2 groups in our study, consistent with the previously reports above. In our survival analysis where recurrence free survival time was assessed, results showed that both procedures had the same prognosis potential, and this agrees with the previous published reports [1,18]. The results of this study and the described recent studies suggest that at least for current tumors, which may be smaller and of a different histologic type than in earlier eras, thoracoscopic segmentectomy may be an acceptable operation from an oncologic standpoint. Moreover, as many as 11.5% of patients undergoing surgery for lung cancer have additional primary cancers develop within their lifetimes and thus require additional resection [34]. Limited pulmonary resection allows future resections through the preservation of lung volume.

So far there are few reports about VATS bilateral resection and most of the reported operations were for bilateral lobectomy or bilateral wedge resection [35,36] and no data have been reported on bilateral segmentectomy to our knowledge. Among those 3 patients in our study who received multiple segmentectomies, 2 underwent bilateral segmentectomies and bilateral primary lesions were found in both patients. They are free of recurrence and well now. Our successful resection of bilateral segmentectomy has expanded the capability of VATS thoracic surgeons with a new list of resectable segments and no doubt more patients will benefit from the technical advance when lobectomy is not an option due to compromised lung function.

Our results from multivariate Cox regression model analysis were consistent with those from other reports [1,18]. The two resection procedures as well as histopathologic types were not related to recurrence rates as risk factors. Although contrary to the general acknowledge tumor stage and tumor size were not found to be indicators of recurrence, this may well be because of small number of recurrence observed due to relatively short follow up.

The limitations of this study lie in the retrospective nature of the study where the selection of patients was not random and patient selection bias is likely. As a consequence, the inherent differences in the tumor size and follow-up period between VATS segmentectomy and VATS lobectomy are difficult to address objectively. Relatively short follow up periods and thus small number of recurrences and mortalities may reduce the power to detect difference if any between the two procedures. We plan to continue to closely follow up on these patients.

## Conclusions

VATS segmentectomy is a safe and equally effective option for selected patients of stage I NSCLC if performed by experienced thoracoscopic surgeons. With experience accumulated, minimally invasive strategies can be applied to more challenging operations, such as bilateral segmentectomy or in patients with compromised lung function where lobectomy is not an option.

## Abbreviations

NSCLC: Non-small lung cancer; VATS: Video-assisted thoracic surgery; C-VATS: Complete-VATS; TDT: Tumor doubling time; ADC: Adenocarcinoma; BAC: Bronchiolo-alveolar carcinoma; LCSG: Lung cancer study group.

## Competing interests

The authors declare that they have no competing interests.

## Authors’ contributions

ZXJ conducted the study and drafted the manuscript. QLQ collected the data and performed the statistical analysis. LQQ conceived of and supervised the study, and HJ participated in its design and coordination and helped to draft the manuscript. All authors read and approved the final manuscript.

## Authors’ information

Liqiang Qian is Co-first author.
